# Oxygen saturation as a predictor of mortality in hospitalized adult patients with COVID-19 in a public hospital in Lima, Peru

**DOI:** 10.1371/journal.pone.0244171

**Published:** 2020-12-28

**Authors:** Fernando Mejía, Carlos Medina, Enrique Cornejo, Enrique Morello, Sergio Vásquez, Jorge Alave, Alvaro Schwalb, Germán Málaga

**Affiliations:** 1 Instituto de Medicina Tropical Alexander von Humboldt, Universidad Peruana Cayetano Heredia, Lima, Peru; 2 Hospital Cayetano Heredia, Lima, Peru; 3 School of Medicine, Universidad Peruana Unión, Lima, Peru; Osaka University Graduate School of Medicine, JAPAN

## Abstract

**Introduction:**

Peru is among the top ten countries with the highest number of coronavirus disease 2019 (COVID-19) cases worldwide. The aim of the study was to describe the clinical features of hospitalized adult patients with COVID-19 and to determine the prognostic factors associated with in-hospital mortality.

**Methods:**

We conducted a retrospective cohort study among adult patients with COVID-19 admitted to Hospital Cayetano Heredia; a tertiary care hospital in Lima, Peru. The primary outcome was in-hospital mortality. Multivariate Cox proportional hazards regression was used to identify factors independently associated with in-hospital mortality.

**Results:**

A total of 369 patients (median age 59 years [IQR:49–68]; 241 (65.31%) male) were included. Most patients (68.56%) reported at least one comorbidity; more frequently: obesity (42.55%), diabetes mellitus (21.95%), and hypertension (21.68%). The median duration of symptoms prior to hospital admission was 7 days (IQR: 5–10). Reported in-hospital mortality was 49.59%. By multiple Cox regression, oxygen saturation (SaO_2_) values of less than 90% on admission correlated with mortality, presenting 1.86 (95%CI: 1.02–3.39), 4.44 (95%CI: 2.46–8.02) and 7.74 (95%CI: 4.54–13.19) times greater risk of death for SaO_2_ of 89–85%, 84–80% and <80%, respectively, when compared to patients with SaO_2_ >90%. Additionally, age >60 years was associated with 1.88 times greater mortality.

**Conclusions:**

Oxygen saturation below 90% on admission is a strong predictor of in-hospital mortality in patients with COVID-19. In settings with limited resources, efforts to reduce mortality in COVID-19 should focus on early identification of hypoxemia and timely access to hospital care.

## Introduction

In December 2019, the city of Wuhan, China, became the center of an outbreak of atypical pneumonia caused by the severe acute respiratory syndrome coronavirus 2 (SARS-CoV-2) [1]. The cases of coronavirus disease (COVID-19) spread rapidly throughout China and other countries and the outbreak was declared a pandemic by the World Health Organization (WHO) on March 11, 2020.

Most patients with COVID-19 only experience mild symptoms, but some develop a more severe disease that requires hospitalization. Approximately 14.2% to 30% of in-patients are further admitted to the ICU, primarily for mechanical ventilation [2, 3]. The mortality in hospitalized COVID-19 patients ranges from 13.2% to 28.3%; with the majority of reports coming from China and United States of America (USA) [3–7]. However, very little information is available regarding in-hospital mortality and its prognostic factors in Latin America [8, 9].

From March 6, 2020, when the first COVID-19 case was reported in Peru, up to July 22, 2020, there have been 366,550 reported cases and 17,455 deaths (Case fatality rate: 4.76%) [10]. In order to mitigate the pandemic impact, the Peruvian Ministry of Health took numerous measures, like declaring a state of national emergency, designating hospitals exclusively for COVID-19 care, telemedicine implementation, increasing diagnostic testing with serological rapid diagnostic tests (RDT) and recommending treatments involving drugs with limited clinical evidence, like ivermectin. Despite these interventions, Peru is among the top ten countries with the highest number of reported cases worldwide with a total excess of deaths of 149% compared to recent years [11].

In order to understand the demographic and clinical characteristics of hospitalized patients with COVID-19 in Peru, as well as to identify prognostic factors for in-hospital mortality, we conducted a retrospective cohort study at a referral teaching hospital in Lima, Peru. Our goal was to identify prognostic factors that would allow for the design of future strategies to optimize the current management of COVID-19 patients in limited resource settings.

## Materials and methods

A retrospective cohort study was conducted. We reviewed paper medical records of adult patients with COVID-19 admitted at Hospital Cayetano Heredia (HCH), a tertiary care referral hospital in the city of Lima, Peru which is at sea level. This public hospital serves approximately three million people, most of them of low-socioeconomic status and including a large number of immigrants, both Peruvians from rural areas and Venezuelan immigrants. During the SARS-CoV-2 pandemic, the hospital implemented 241 beds for COVID-19 care, including 23 ICU beds. The study was approved by the Research Ethics Committee of Hospital Cayetano Heredia (Approval code: 059–2020). Permission was granted to review all available records on the archive of hospitalized COVID-19 patients. Patient consent was not required as data gathered did not contain patient identifiable information.

Confirmed COVID-19 cases by a positive test for SARS-CoV-2, as well as probable cases based on clinical presentation and imaging, were included. Laboratory testing for SARS-CoV-2 included either positive IgM and/or IgG in serologic RDT, or molecular testing using reverse transcriptase polymerase chain reaction (RT-PCR) analysis.

The paper medical records were archived after patient discharge, transfer or death. We reviewed all medical records that were available at HCH archive from March 29 to June 11, 2020; data on patients that were hospitalized by the latter date were not included as the medical records were still in use. The information was collected using an electronic data collection form created on the Open Data Kit (ODK) Collect platform (Get ODK Inc., California, USA). Patient information included demographics, comorbidities, vital signs on admission, laboratory results, and hospitalization outcomes: length of stay, ICU admission, discharge, or death. Patients were excluded from the study if they were transferred to another facility or if they left the hospital against medical advice (AMA). Obesity was not determined by measuring weight and height, but rather by the patient’s self-reported weight and height or by treating physician’s criteria. Tachypnea was defined as ≥22 breaths per minute, and acute respiratory distress syndrome (ARDS) was defined using the pulse oximetry saturation/fraction of inspired oxygen (SaO_2_/FiO_2_) ratio threshold of <235.

Statistical analysis was performed using Stata SE 16.1 (StataCorp., Texas, USA). Categorical variables were reported as frequencies and percentages, and continuous variables as medians with interquartile range (IQR). Comparisons were made between COVID-19 patients who were discharged from hospital or died by bivariate analysis: the Chi-square test and Fisher’s exact test were used for categorical variables while the Mann-Whitney U test and Kruskal-Wallis test were used for continuous variables, where a 2-sided p value <0.05 was considered statistically significant. Hazard ratios (HR) and its 95% confidence intervals (CI) were calculated using Cox proportional hazards regression to assess the association between baseline factors at admission and in-hospital mortality. Multivariate Cox regression model was constructed to evaluate the association of demographic characteristics, comorbidities and SaO_2_ on admission with in-hospital mortality; variables were chosen via forward stepwise selection by statistical significance in the bivariate analysis and clinical relevance. Additionally, a secondary analysis was performed to include tachypnea on admission (replacing SaO_2_) in the multivariate Cox regression model for in-hospital mortality. The proportional hazard assumptions were checked using Schoenfeld residuals.

## Results

**Fig 1:**
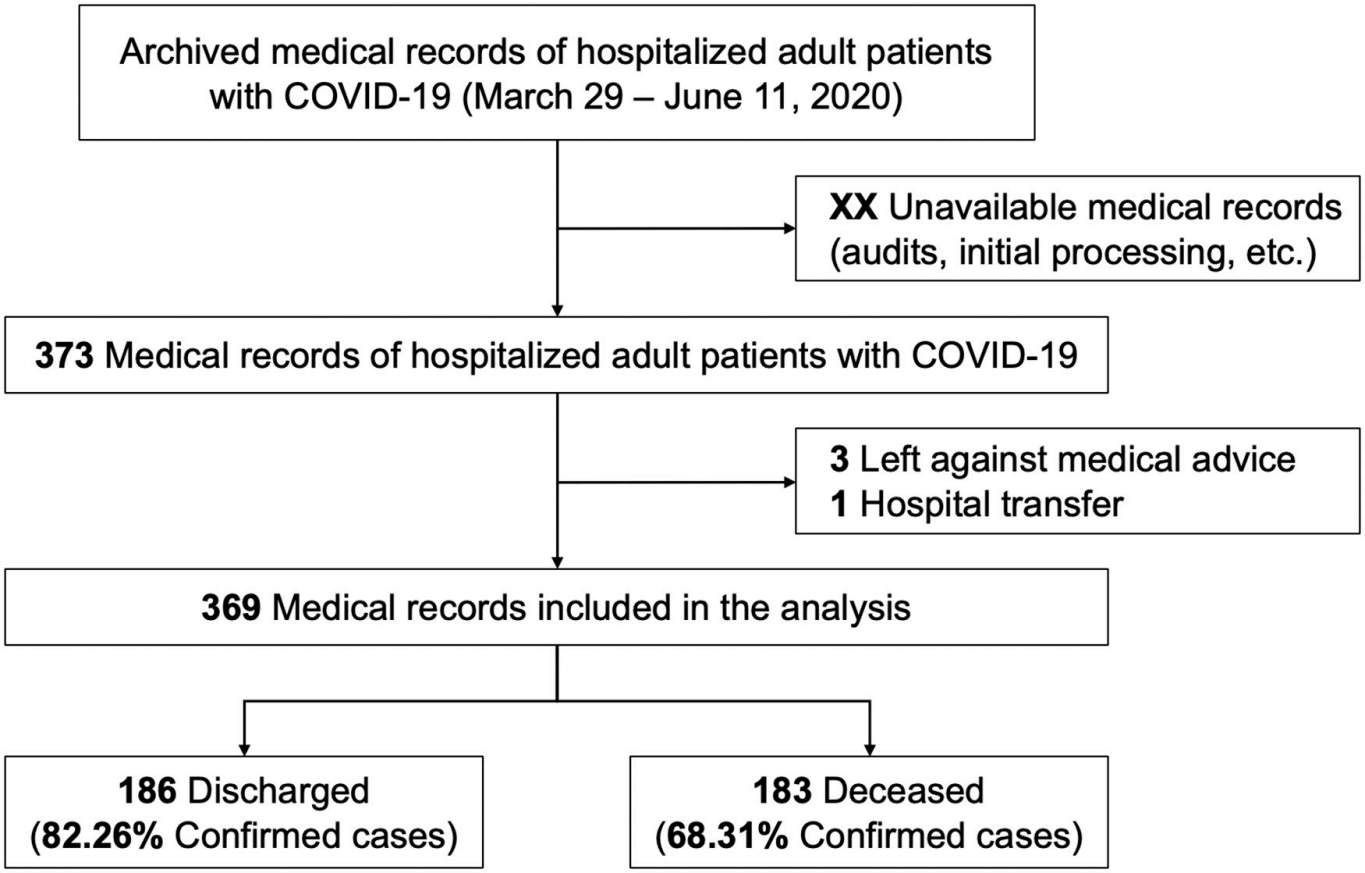
Flowchart of adult hospitalized patients included in the cohort and their outcome.

A total of 373 medical records of adult patients with COVID-19 admitted at HCH were identified; four patients were excluded as they were transferred or left AMA (Fig 1). Of the 369 patients included in the analysis, 241 (65.31%) were male and the median age was 59 years (IQR: 49–68). Most patients (68.56%) had at least one comorbidity: obesity (42.55%), diabetes mellitus (21.95%) and hypertension (21.68%). A total of 278 (75.34%) were confirmed COVID-19 cases, 250 (89.93%) by a positive serological RDT, 16 (5.75%) by a positive RT-PCR, and 12 (4.32%) by both methods. The median duration of symptoms prior to hospitalization was 7 days (IQR: 5–10) (Table 1).

**Table 1:** Characteristics of adult hospitalized patients with COVID-19 at hospital admission.

Characteristic	All (n = 369)	Discharged (n = 186)	Deceased (n = 183)	P
**Demographic characteristics**				
Age, years, median (IQR)	59 (49–68)	54 (44–62)	65 (57–74)	<0.001
Age ≥60 years	183 (49.59)	56 (30.11)	127 (69.40)	<0.001
Male	241 (65.31)	121 (65.05)	120 (65.57)	0.916
Underlying comorbidities	253 (68.56)	115 (61.83)	138 (75.41)	0.005
Obesity	157 (42.55)	66 (35.48)	91 (49.73)	0.006
Diabetes	81 (21.95)	35 (18.82)	46 (25.14)	0.143
Hypertension	80 (21.68)	26 (13.98)	54 (29.51)	<0.001
Asthma	24 (6.50)	16 (8.60)	8 (4.37)	0.099
Prior tuberculosis disease	15 (4.07)	12 (6.45)	3 (1.64)	0.017*
Cancer	9 (2.44)	5 (2.69)	4 (2.19)	0.510*
Chronic kidney disease	8 (2.17)	2 (1.08)	6 (3.28)	0.137*
Acute myocardial infarction	6 (1.63)	3 (1.61)	3 (1.64)	0.650*
Others	11 (2.95)	2 (1.08)	9 (4.92)	0.029*
Symptom onset before admission, days, median (IQR)	7 (5–10)	7 (6–10)	7 (5–10)	0.3458
**Vital signs**				
Temperature, °C, median (IQR)	36 (36–37)	37 (36–37)	36 (36–37)	0.797
Respiratory rate, breaths/min, median (IQR)	28 (25–32)	26.5 (23–30)	30 (26–35)	<0.001
Tachypnea (≥22 breaths/min)	339 (91.87)	159 (85.48)	180 (98.36)	<0.001
Heart rate, beats/min, median (IQR)	108 (96–118)	103.5 (94–113)	110 (98–120)	<0.001
Systolic blood pressure, mmHg (n = 368), median (IQR)	110 (100–120)	110 (100–120)	110 (100–130)	0.019
Diastolic blood pressure, mmHg (n = 367), median (IQR)	70 (60–80)	70 (60–80)	70 (68–80)	0.192
Oxygen saturation by pulse oximetry, %, median (IQR)	87 (77–92)	91 (88–94)	78 (67–85)	<0.001
≥90%	131 (35.50)	112 (60.22)	19 (10.38)	<0.001
89–85%	85 (23.04)	56 (30.11)	29 (15.85)	
84–80%	48 (13.01)	14 (7.53)	34 (18.58)	
<80%	105 (28.46)	4 (2.15)	101 (55.19)	
ARDS (SaO_2_/FiO_2_ <235)	9 (2.44)	0	9 (2.44)	0.002*
**Laboratory evaluations, median (IQR)**				
Hematocrit, % (n = 331)	41 (37–44)	41 (38–45)	40 (36–44)	0.062
White blood cell count, cells/mmˆ3 (n = 330)	11,565 (8,260–15,270)	10,200 (7,470–13,370)	12,860 (9,130–16,600)	<0.001
Absolute lymphocyte count, cells/mmˆ3 (n = 330)	926.9 (675.9–1380.75)	1143 (783–1511.1)	792.25 (570.5–1168)	<0.001
Leukocytosis and relative lymphopenia (<10%) (n = 204)	156 (76.47)	54 (63.53)	102 (85.71)	<0.001
Platelets, cells/10ˆ9/L (n = 327)	291 (223–364)	308.5 (245–387)	265 (200–350)	<0.001
AST, U/L (n = 258)	54 (37–85)	54 (34–85)	54 (41–84)	0.381
ALT, U/L (n = 255)	51 (32–89)	63 (41–100)	44 (29–75)	0.002
Urea, mg/dL (n = 311)	36 (25–52)	30 (22–41)	42 (29–60)	<0.001
Creatinine, mg/dL (n = 317)	0.7 (0.5–0.9)	0.7 (0.5–0.8)	0.8 (0.6–1.0)	0.006
Sodium, mmol/L (n = 282)	138 (135–140)	138 (135–140)	138 (135–140)	0.815
Potassium, mmol/L (n = 282)	4.4 (3.9–4.6)	4.3 (3.9–4.6)	4.4 (3.9–4.7)	0.654
C-reactive protein, mg/dL (n = 250)	96 (48–192)	92 (48–96)	96 (92–192)	<0.001
Lactate dehydrogenase, U/L (n = 262)	469.5 (356–658)	381 (310–471)	611 (460–764)	<0.001
Ferritin, ng/mL (n = 78)	777.5 (449–1,707)	669 (359–1,325)	1,267 (640–2,227)	0.005
**During hospital stay**				
Length of stay, days, median (IQR)	7 (3–10)	8 (5–12)	5 (2–8)	<0.001
High-flow oxygen requirement (FiO_2_ ≥0.36)	268 (72.63)	87 (46.77)	181 (98.91)	<0.001
ICU requirement (FiO_2_ 0.8)	226 (61.25)	49 (26.34)	177 (96.72)	<0.001
ICU admission (n = 226)	23 (10.18)	6 (12.24)	17 (9.60)	0.589
ARDS (SaO_2_/FiO_2_ <235)	248 (67.21)	67 (36.02)	181 (98.91)	<0.001

Values are n (%) unless noted otherwise.

*Fisher’s exact test.

IQR: Interquartile range; ARDS: Acute respiratory distress syndrome; SaO_2_: Oxygen saturation. FiO_2_: Fraction of inspired oxygen; AST: Aspartate aminotransferase; ALT: Alanine aminotransferase; ICU: Intensive care unit.

Respiratory and heart rates were elevated on admission, with a median of 28 breaths and 108 beats per minute (IQR: 25–32 and 96–118, respectively). Tachypnea was present in 91.27%. Additionally, SaO_2_ on admission was generally low with a median of 87% (IQR: 77–92), and more than a quarter of the patients (28.46%) were admitted with SaO_2_ below 80%. The leukocyte count was elevated (11,565 cells/mm^3^, IQR: 8,260–15,270), and 76.47% of patients had leukocytosis with associated relative lymphopenia (<10% of the leukocyte count). Laboratory parameters of severe COVID-19, such as C-reactive protein (median 96 mg/dL, IQR: 48–192) and lactate dehydrogenase (median 469.5 U/L, IQR: 356–658) were also elevated. Most patients (72.63%) required high-flow oxygen therapy (FiO_2_ ≥0.36); which included 226 (61.25%) that required use of a non-rebreather (NRB) mask at 15 L/min. Only 10.18% of patients on a NRB mask entered the ICU. Additionally, 67.21% of patients developed ARDS by SaO_2_/FiO_2_ ratio during hospitalization. The median hospital stay before discharge was 8 days (IQR: 5–12), with death generally occurring on day 5 of hospitalization (IQR: 2–8). For discharged patients, the median length of stay was inversely proportional to SaO_2_ values on admission: 7 days (IQR: 4–10) for SaO_2_ ≥90%, 9.5 days (IQR: 6–13.5) for SaO_2_ 89–85%, 10.5 days (IQR: 8–14) for SaO_2_ 84–80%, and 22 days (IQR: 17.5–22.5) for SaO_2_ <80% (p <0.001).

**Fig 2:**
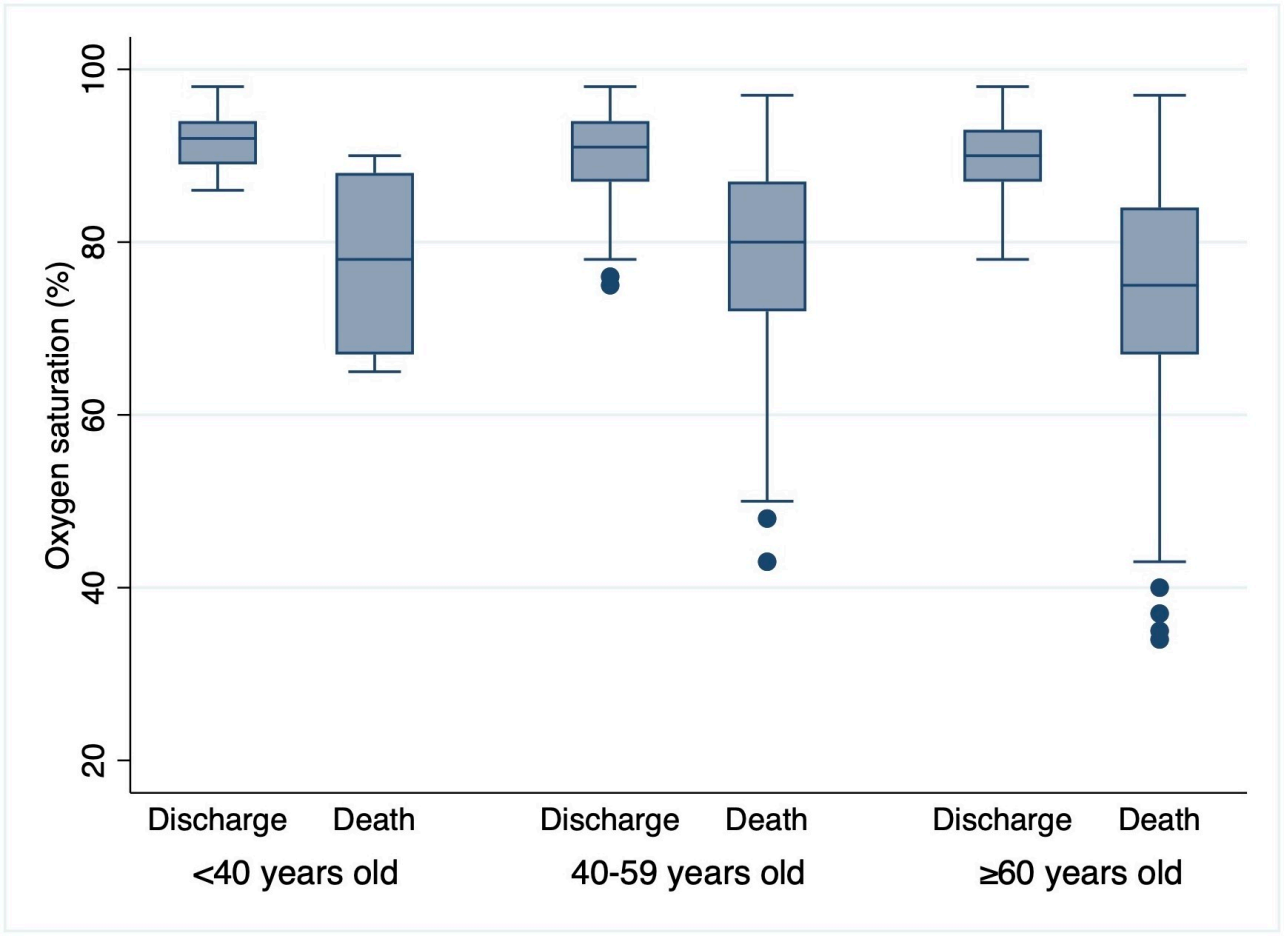
Box plot comparing oxygen saturation medians by age group and by outcome.

One half of patients (183, 49.59%) died during hospitalization. Significant differences were observed in the proportion of patients aged ≥60 years and with comorbidities, mainly obesity and hypertension, with higher frequencies in the deceased group. The SaO_2_ value on admission was lower in deceased patients compared to discharged patients (78% vs. 91%, p <0.001). This difference was maintained for each SatO_2_ category and was also constant between different age groups (Fig 2). The proportion of ARDS on admission or developed during hospitalization was greater among deceased participants. Lower absolute lymphocyte counts, higher white blood cell counts, and higher levels of C-reactive protein and lactate dehydrogenase were found among deceased participants.

**Fig 3:**
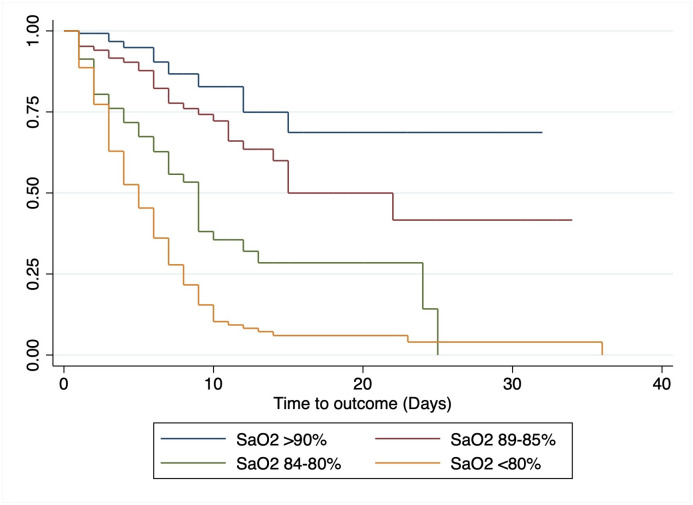
Kaplan-Meier survival curve by different oxygen saturation category at admission.

Predictive factors of in-hospital mortality were evaluated using Cox proportional hazards regression. Bivariate analysis found that all SaO_2_ categories <90% on admission were associated with a hazard mortality from 1.93 (95%CI: 1.07–3.49) to 9.13 (95%CI: 5.50–15.14) times higher compared to SaO_2_ ≥90%. Survival analysis based on SaO_2_ categories was plotted using Kaplan-Meier curves (Fig 3). In the bivariate analysis, age ≥60 years (cHR 2.83; 95%CI: 2.03–3.93) and hypertension (cHR 1.63; 95%CI: 1.18–2.26) were also associated with in-hospital mortality. In the multivariate analysis, after adjusting for male sex, age ≥60 years and comorbidities (obesity, diabetes, and hypertension), SaO_2_ values of less than 90% correlated independently with in-hospital mortality, presenting 1.86 (95%CI: 1.02–3.39), 4.44 (95%CI: 2.46–8.02) and 7.74 (95%CI: 4.54–13.19) times greater risk of death for SaO_2_ of 89–85%, 84–80% and <80%, respectively. Likewise, age ≥60 years was independently associated with in-hospital mortality (aHR 1.88; 95%CI: 1.32–2.69) (Table 2). After secondary analysis, tachypnea was associated with in-hospital mortality both in the bivariate analysis (cHR 4.82; 95%CI: 1.54–15.12) and in the multivariate model when replacing SaO_2_ categories (aHR 3.99; 95%CI: 1.27–12.54).

**Table 2:** Baseline predictor factors of mortality in adult hospitalized patients with COVID-19.

Characteristic	cHR (95%CI)	aHR (95%CI)*
Age ≥60 years	2.83 (2.03–3.93)	1.88 (1.32–2.69)
Male	0.91 (0.66–1.25)	0.81 (0.58–1.13)
Obesity	1.16 (0.86–1.57)	0.99 (0.72–1.35)
Diabetes	1.19 (0.84–1.69)	1.10 (0.77–1.59)
Hypertension	1.63 (1.18–2.26)	1.20 (0.85–1.70)
Oxygen saturation		
SaO_2_ ≥90%	Ref.	Ref.
SaO_2_ 89–85%	1.93 (1.07–3.49)	1.86 (1.02–3.39)
SaO_2_ 84–80%	4.71 (2.65–8.40)	4.51 (2.49–8.12)
SaO_2_ <80%	9.13 (5.50–15.14)	7.81 (4.63–13.17)

cHR: Crude hazard ratios; aHRa: Adjusted hazard ratios; CI: Confidence interval; SaO_2_: Oxygen saturation. Patient in-hospital mortality was adjusted for male sex, age ≥60 years, obesity, diabetes, hypertension, and SaO2 value categories of less than 90%.

*n = 357.

## Discussion

This study serves as the first report of hospitalized COVID-19 patients in Peru. Unlike other published cohorts, our study presents data from a middle-income country in Latin America, that prior to the SARS-CoV-2 pandemic faced the challenges of a fragmented healthcare system, overcrowding of hospitals, and poor health expenditure (5% of the gross domestic product) [12]. Our primary care system is deficient in infrastructure and resources and so far has been unable to play a leading role in patient care during this pandemic [13, 14]. Our findings should be interpreted within this context.

Our study population shares similar characteristics with other cohorts [15, 16]. The most frequent comorbidities were obesity, diabetes mellitus and hypertension, all of them associated with severe illness and/or mortality in patients with COVID-19 [17]. There was a higher frequency of obesity in the deceased group, which is consistent with previous reports, where obesity was found to be a risk factor for ICU admission and mechanical ventilation [18]. Also, certain conditions associated with obesity, like diabetes mellitus and dyslipidemia, where previously shown to have higher mortality in patients with COVID-19 [19]. Therefore, the presence of these comorbidities contribute to a higher demand of ICU care among hospitalized patients. Unfortunately, the Peruvian public healthcare system was not prepared to satisfy this demand, with only 0.2 ICU beds for every 10,000 inhabitants in Peru at the beginning of the pandemic [20].

One of the most striking findings of our study is the elevated in-hospital mortality; 49.6%. This mortality rate is much higher than in other middle- and high-income countries like China and USA, between 13.2% and 28.3% [3–7]. The same is true for other middle-income Latin American countries, like Brazil and Mexico, with in-hospital mortality between 16.9 and 21.9% [8, 9]. In contrast, a similar mortality rate (42.8%) among hospitalized patients was reported in Honduras, where the median age of fatal COVID-19 cases was 62 years [21]. We have not found reports of in-hospital mortality in COVID-19 from low-income countries. Interestingly, unpublished data suggests that mortality from COVID-19 in private hospitals in Lima would not differ from high-income countries. Therefore, the higher mortality in our cohort from a public hospital could be associated with economic and social barriers for access to healthcare in Peru, as well as the amount of resources available for inpatient care [22].

The degree of hypoxemia (principally SaO_2_ <85%) on admission was independently associated with in-hospital mortality in our cohort. Hypoxemia on admission as a predictor of in-hospital mortality was previously described in cohorts from China and USA [6, 7, 23]. However, to our knowledge, this is the first study from Latin America to identify a similar association. *Petrilli et al*. found SaO_2_ <88% on admission was associated with mortality, although the proportion of patients with hypoxemia was lower than in our cohort (15.5% vs. 64.5%, respectively), correlating with lower in-hospital mortality (24.3%) [6]. These findings could suggest that a high proportion of patients from our cohort were hospitalized too late, after developing significant hypoxemia and resulting in higher mortality. Since the median time of symptom onset was similar for both groups, one could hypothesize that some of the patients in the diseased group developed hypoxemia faster prior to hospitalization. This implies that we need a better, faster way to recognize hypoxemia in the community setting, which becomes challenging in the context of the “silent hypoxemia” that many COVID-19 patients experience early in the course of disease [24]. Additionally, some of our patients experienced further delay in hospitalization, as they previously visited other facilities where they were unable to provide adequate support and oxygen therapy due to lack of resources. The importance of hypoxemia as a predictor of mortality goes beyond COVID-19 and involves other lower respiratory infections as well, as previously studied in children [25].

It comes as no surprise that median SaO_2_ levels on admission are significantly lower in our cohort compared with others published [2, 6]. This suggests late presentation, either due to lack of awareness of alarm signs, lack of access to transportation, fear of going to a hospital, and other limitations to timely access to emergency medical care. Consequently, it is essential to encourage patients with suspected COVID-19 to attend the hospital early in the course of disease. In this regard, strategies such as the one implemented in Colombia, where patients receive pulse oximeters to monitor for hypoxemia while ambulatory, represent enticing low-risk interventions that could help with timely detection of hypoxemia. Pulse oximeters are now relatively inexpensive (between 30 to 40 US dollars in Peru) and can be made widely available in primary care facilities and in the community. This strategy already seems to be helping in the identification of decompensating outpatients with COVID-19 in USA to come to the hospital early for oxygen therapy [26]. For settings where implementation of pulse oximeter use in the community would not be feasible, measuring tachypnea could be an alternative predictive factor on mortality, one that is also simple and inexpensive, as shown in our additional exploratory analysis. Additionally, our findings raise the need for more hospital beds and increased availability of supplemental oxygen as a matter of public health priority; upgrades necessary to meet the expected increase in demand for early care of hypoxemia. These upgrades will need to be complemented with a robust call center and ambulance system in which patients with alarm signs are quickly identified and transferred to the hospital.

Hypoxia and inflammation are intertwined at the molecular, cellular, and clinical level [27]. Clinical events that produce acute hypoxemia enhance various cytotoxic functions of neutrophils and can promote hyperinflammation [27]. Animal models found that exposure to low oxygen concentrations results in increased vascular permeability, accumulation of inflammatory cells, and elevated serum cytokine levels [27]. Thus, hypoxia not only represents a consequence of respiratory disease, but also contributes significantly to progressive lung damage after establishment of the initial injury.

One of the main complications of COVID-19 is ARDS, developing 8 days after symptom onset [28]. The greater the degree of hypoxemia or the delay in intubation, the greater the mortality in ARDS (27–45%) [29]. The median time from symptom onset in our cohort was 8 days and 98.9% of our deceased patients developed ARDS during hospitalization, which places ARDS as one of the main physiological processes suspected behind our high in-hospital mortality.

It is important to note that, according to the level of hypoxemia described, most of our patients would qualify as critical on admission, in which case the mortality is similar to the one described by *Wu & Mcgoogan* (49%) [30]. In our cohort, 61.3% of patients required ICU care based on high-flow oxygen requirement (FiO_2_ ≥ 0.8). Of these, only 10.2% were admitted to the ICU, which is extremely low. Not providing required ICU care in a timely fashion could have directly influenced in-hospital mortality. However, if one considers the possibility of some of our critically ill patients arriving at the hospital with prolonged hypoxemia, one could not rule out that their fatal outcome may have been unchanged even if they had access to ICU care. This would rely on the fact that the higher the hypoxemia, the higher the mortality in ARDS and also that tissue damage due to hypoxia may sometimes reach a “point of no return”, where patients with respiratory failure are refractory to standard critical care interventions [31].

Augmenting ICU capacity in our setting would have saved more lives. However, we should consider that ICU length of stay is two weeks on average for patients with COVID-19, which means that each ICU bed may be able to accommodate two patients per month. Furthermore, with ICU estimated mortality around 50%, we can assume that, for each ICU bed that we implement, we may save the life of one additional patient per month [16, 32]. In the context of a healthcare system with limited economic and human resources, further strained by the current pandemic, we believe that investing resources towards increasing hospitalization capacity for early general care and oxygen therapy, as well as empowering the primary healthcare system to early detect hypoxemia in the community would probably have a bigger impact on mortality than allocating too many resources on implementing more ICU beds. The fact that we found an inverse relationship between lower SaO_2_ on admission and longer hospital length of stay further supports the notion that the strategies discussed would be more cost-effective.

The main strength of our study is that our population and setting are representative of care for COVID-19 patients at a public hospital in Lima, the city with the most cases in Peru [10]. Our cohort design allows for obtaining adequate information on mortality risk on admission, taking into consideration clinical and demographic factors that may be applicable to any healthcare setting. It is also the first study to evaluate predictive factors of mortality in our country and the first one to associate hypoxemia on admission with inpatient mortality in Latin America. We hope our findings contribute as a framework for designing patient-care strategies for COVID-19 that are adapted to the challenges of low- and middle-income countries.

The main limitations of our study are related to the nature of retrospective data collection. Data recorded on medical records was that which was deemed relevant by the treating physician. However, we selected many variables that are considered relevant in the management of patients with COVID-19 and therefore recorded in all cases, allowing us to gather enough data to perform analysis with a high number of observations. Additionally, some records were not available for review by June 11, 2020, as they were being audited, retained prior to archiving, or still in use for patients still admitted to the hospital. The absence of this information could introduce a source of bias, although we estimate that the proportion of records meeting these criteria was small. Due to the method used to determine obesity it is possible that the number of obese patients in our cohort might have been underestimated and thus affected its association with mortality. Unlike other cohorts, the number of RT-PCRs performed among our patients is low, with the majority of cases being confirmed by means of a positive serologic RDT. Nevertheless, these serologic RDT have a specificity of 98.9% and their sensitivity increases to 70% in patients with more than7 days of symptoms, as is the case for the majority of our cohort [33]. Having evaluated patients with typical disease presentation and course in the context of an active pandemic, we consider the positive predictive value to be high.

## Conclusions

In conclusion, among patients with COVID-19 who were admitted to a public hospital in Peru, in-hospital mortality was high and was independently associated with oxygen saturation below 90% on admission and with age over 60 years. Early identification of hypoxemia and transfer to a healthcare facility with oxygen access should be the goal of outpatient monitoring strategies for suspected cases of COVID-19, so timely access to care helps prevent the deleterious effects of persistent hypoxemia and, in turn, reduce in-hospital mortality.

## Supporting information

S1 File Study database. Recollected data of hospitalized COVID-19 patients in HCH.
(DTA)
